# The Case of the Lime-green Stool: A Case Report and Review of Occult Blood Testing in the Emergency Department

**DOI:** 10.5811/cpcem.2021.4.51656

**Published:** 2021-05-25

**Authors:** James D. Salisbury, Jennifer G. Goodrich, Nicholas M. McManus, Ryan P. Offman

**Affiliations:** Mercy Health, Department of Emergency Medicine, Muskegon, Michigan

**Keywords:** Melena, food coloring, fecal occult blood test, gastrointestinal hemorrhage, Glasgow-Blatchford Score

## Abstract

**Introduction:**

Food dyes mimicking gastrointestinal (GI) hemorrhage have been described in literature. However, reports of food additives causing melanotic stools and falsely positive fecal occult blood tests (FOBT) are uncommon in literature.

**Case Report:**

We present a case of a 93-year-old with FOBT positive melanotic stool, felt to be falsely positive due to food additives.

**Conclusion:**

Evaluation for GI bleeding accounts for 0.3% of yearly visits to the emergency department (ED).^1^ While FOBT is commonly used, its clinical validity in the ED is not supported by guidelines. We showcase the limitations of the FOBT and review the causes of false positive FOBT.

## INTRODUCTION

Naturally derived food dyes using annatto extract, beta-carotene, beet powder, caramel color, paprika, saffron, turmeric, and fruit and vegetable juices have been used in food preparation for centuries. Synthetic color additives were first discovered in 1856 and gained popularity in food and cosmetic production in the early 1900s.^2^ While color additives are approved for human consumption by the US Food and Drug Administration due to a lack of scientific evidence of direct harm to consumers, the secondary effects of food dyes are still in question.^2^ Since the explosion of fruit-flavored children’s cereals in the 1970s, cases of red-colored stool prompting evaluation of gastrointestinal (GI) bleeding have been documented in the medical literature.^3^ Further, certain foods such as those with animal-derived heme, green vegetables, broccoli, cauliflower, cantaloupe, carrots, squash, figs, horseradish, grapefruit, melons, tomatoes, pumpkins, and gourds have all been documented to cause falsely positive chemical fecal occult blood tests (FOBT).^4^

Concern for stool color abnormalities is frequently encountered by the emergency physician. While the true clinical consequences of falsely positive FOBT are unknown, the downstream consequences of over-diagnosis should be considered. We present a case of an under-reported scenario with potential clinical implications that highlights both the limitations of the FOBT and the importance of a detailed history to assist in the diagnosis of pathology and to protect our patients from the risks of over-testing.

## CASE REPORT

A 93-year-old female presented to the ED with complaints of two episodes of black, tarry stool over the previous 24 hours that turned lime-green in color when it contacted the water in the toilet bowl. She expressed personal concern for GI hemorrhage, as she had a similar episode of melena one year prior and was subsequently found to have a gastric ulcer thought to be secondary to non-steroidal anti-inflammatory drug (NSAID) use. The ulcer was treated with endoscopic ablation and the patient had been symptom free since that time. Further past medical history included hypertension and stage III chronic kidney disease. Her only home medications included a daily multivitamin and an omega-3 polyunsaturated fatty acid supplement.

A detailed history obtained in the ED revealed that she had eaten a fast-food hamburger and frozen beverage the evening prior to the onset of her perceived melanotic stool. Further questioning revealed that the hamburger bun and beverage were both dyed black in color as a promotional event by the fast-food restaurant in celebration of Halloween. She denied ongoing NSAID use that had seemingly contributed to her previously mild upper GI bleed (UGIB).

The patient was hemodynamically stable with a blood pressure of 132/79 millimeters of mercury, a heart rate of 74 beats per minute, a respiratory rate of 16 breaths per minute, and an oxygen saturation of 96% on room air. She denied abdominal discomfort. Serology testing showed a chronic and otherwise stable hemoglobin of 11.0 grams per deciliter (g/dL) (reference range 11.5–15.5 g/dL), a blood urea nitrogen (BUN) of 17 milligrams per deciliter (mg/dL) (8–28 mg/dL), a creatinine of 1.8 mg/dL (0.5–1.5 mg/dL), and a BUN to creatinine ratio of 9.4.

Digital rectal exam done in the ED revealed a bright, lime-green stool without obvious evidence of melena or hematochezia. A guaiac FOBT (gFOBT) was performed. Despite the grossly bright, lime-green appearance of the specimen, this turned dark green to black in color with a blue hue when developer was introduced. Appearance of occult blood in stool specimens is considered when a blue coloration is appreciated on the indicator card. However, given the duration of the patient’s symptoms, the absence of acute anemia, the lack of observed melena on digital rectal exam, and the history of recent ingestion of artificially dyed food, a false positive gFOBT was considered.

A shared decision-making discussion on inpatient observation vs close outpatient follow-up was done with the gastroenterologist, the emergency physician, and the patient. Given the absence of melena on digital rectal exam, a stable hemoglobin level near her baseline, and patient preference, she was felt to be reasonable for close outpatient follow-up. Further, she had a calculated Glasgow-Blatchford Bleeding Score of 1, which would suggest a predicted survivability without intervention at a sensitivity of 98.6%.^6^ Following a four-hour observation period, she remained asymptomatic and without observed melena. The patient was discharged home with close outpatient follow-up the next day.

A repeat hemoglobin level obtained 12 hours after ED discharge was unchanged at 11.2 g/dL. A one-year follow-up of the patient’s medical record from her primary physician showed no return visits to the ED, and no diagnostic or therapeutic colonoscopy had been performed. Her hemoglobin levels remained stable within her baseline range of 11.0 to 12.3 g/dL in the one-year period since ED discharge.

**CPC-EM Capsule**

What do we already know about this clinical entity?

*Evaluation for gastrointestinal bleeding accounts for 0.3% of yearly visits to the emergency department (ED), and fecal occult blood testing (FOBT) is frequently used as a screening tool*.

What makes this presentation of disease reportable?

*Our case highlights the potential for food dyes to cause false positive FOBT results and recommends against its use as a screening tool*.

What is the major learning point?

*While FOBT is validated for its use in colorectal cancer screening, it has limited utility as a screening tool in the ED*.

How might this improve emergency medicine practice?

*Emergency clinicians will better understand the limitation of FOBT and the lack of evidence of its superiority to a thorough history and physical exam*.

## DISCUSSION

Our patient presented with the subjective complaint of melanotic stools, which alone carries a likelihood ratio (LR) of 5.1–5.9 for UGIB.^5^ Further clinical findings to support a suspicion of acute UGIB including melena on examination (LR of 25) and a ratio of BUN to creatinine greater than 30 (LR of 7.5), were not present in our patient.^5^

The three most common causes of UGIB are peptic ulcer disease, esophagogastric varices, and erosive esophagitis.^5^ A strong clinical history to include all medications, supplements, and ingested foods is paramount to assist with supporting or opposing a diagnosis of GI bleed, and patients should be risk stratified based on all available factors of the history and physical exam. In acute GI bleeding, hemoglobin levels may initially falsely appear at baseline values as it takes several hours to be reflected in laboratory specimens and should not be used as a sole predictor of bleeding severity.^5^ The Glasgow-Blatchford Bleeding Score is a risk-stratification tool to assist clinicians in determining who is most likely to need intervention in the setting of UGIB.^6^ A score less than or equal to 1, as was present in our patient, yields a sensitivity and specificity of 98.6% and 34.6%, respectively, and can be used as an adjunctive measurement of those patients who may be reasonable for outpatient evaluation.^6^ However, it is important to understand that a risk-stratification score should not replace clinical evaluation and physician judgment.^5^

While it is recommended that all patients with high clinical concern for UGIB should undergo a diagnostic esophagoduodenoscopy, endoscopy is not a zero-risk procedure, and a risk-to-benefit analysis should be considered on an individual basis.^5^ Serious risks for upper GI endoscopy include bleeding, perforation, infection, and complications of anesthesia, and occur in as many as 0.5% of all cases.^7^ The addition of small-bowel enteroscopy carries a minor and major adverse event rate of 9.1% and 0.7%, respectively.^7^ Significant adverse events of lower endoscopy include bleeding, perforation, and death occurring in 0.02–1% of adult and 1.1–2.4% of pediatric colonoscopies.^8^ In a 2008 cost analysis of management strategies in patients with obscure GI bleeding, patients who did not receive diagnostic intervention were associated with a 59% bleeding cessation rate, while dual-balloon enteroscopy was associated with a fourfold increase in total cost and an 86% bleeding cessation rate.^9^

The FOBT is endorsed by the US Preventive Services Task Force and the American College of Gastroenterology for colorectal cancer screening.^10^ However, its use as a diagnostic tool for suspected acute upper GI bleeding lacks validation in literature.^10^ Three types of fecal occult tests are available: chemical; immunochemical; and deoxyribonucleic acid testing. Deoxyribonucleic acid testing is less commonly available, and clinical utility is limited by its high cost.^4^ Fecal immunochemical tests (FIT) detect human globin, a protein component of hemoglobin and have the advantage of not falsely turning positive from medications, animal hemoglobin, fruit or vegetable compounds.^4^ However, FITs are limited in that they are only sensitive in detecting bleeding from the lower GI tract as globin from upper bleeds are rapidly degraded by proteolytic enzymes, and blood volumes < 100 mL have gone undetected in studies.^4^

In contrast, chemical tests such as the gFOBT are more widely used for bedside testing as they are easy to conduct and comparatively inexpensive. When blood-derived heme is subjected to hydrogen peroxide, oxidation of guaiac acid occurs, and the indicator paper turns blue.^4^ The gFOBT has the advantage of being able to detect small amounts of blood from the upper or lower GI tracts. However, the tests are severely limited by a high rate of false positives with a specificity of only 50% in some studies.^4^ False positive results have been described from ingestion of animal-derived heme, foods with high levels of peroxidase (green vegetables, broccoli, cauliflower, cantaloupe, carrots, squash, figs, horseradish, melons, and pumpkins and gourds), chlorophyll, methylene blue-containing tablets, and blue and blue-green colored tablets at high concentrations.^4^,^12^,^13^

Topical povidone-iodine, which is used as an antiseptic when inserting a Foley catheter, has also been shown to give false positive gFOBT results when the test card is contaminated with this solution.^11^ Further, a false positive result can occur from extra-intestinal sources such as epistaxis, inflammatory conditions such as gastritis, or in the setting of clinically insignificant losses.^10^ Decreased sensitivity of guaiac results have been described with activated charcoal, dimethylaminoethanol, N-acetylcysteine, red chile, red Jell-O, orange juice, Pepto-Bismol, simethicone, spaghetti sauce, red wines, and vitamin C, which inhibits guaiac oxidation.^10^,^13^ False negatives are also possible as is seen with slow or intermittent bleeding.^10^ While iron supplementation has historically been shown to cause a falsely positive gFOBT, more recent in vivo studies have failed to confirm this finding, suggesting iron ingestion will not alter the clinical results of guaiac testing.^14^

Food dyes are well documented in the literature to alter stool color.^3^ However, the direct effect of synthetic food additives on the validity of the FOBT is not apparent in the available literature. When we reviewed the list of ingredients used to make the hamburger bun that had been dyed black and ingested by our patient, we found that the black coloration was achieved using a combination of artificial dyes, including the US Federal Food, Drug and Cosmetic Act-approved Yellow #6, Blue #1, and Red #40.

**Image 1:**
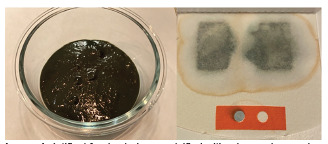
Artifical food coloring emulsified with a burger bun and water (left). Sample of this mixture tested on a guaiac fecal occult blood test (right).

**Image 2:**
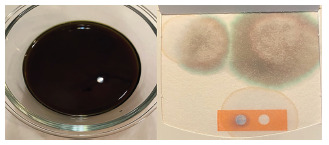
Weak solution of artifical food coloring emulsified with water (left). Sample of this mixture tested on a guaiac fecal occult blood test card (right).

To test our hypothesis that food additives can affect FOBT coloration, a combination of the afore-mentioned dyes was emulsified with a burger bun and water to give the appearance of melena. We then took a sample of this mixture, applied it to our institutional gFOBT, and then applied developer. To our surprise, a blue discoloration similar to the test indicator was observed (Image 1). As a secondary assessment, a weak solution using just water and the three food dyes was tested on a gFOBT card. When the hydrogen peroxide developer was applied, the colors separated with a blue hue moving to the periphery, more similar to what was seen in our case patient (Image 2).

It has been documented that 13% of attending physicians and 15% of resident physicians are unable to accurately interpret FOBT results due to a lack of understanding of result interpretation.^10^,^13^ One can see how this could significantly limit the test’s clinical utility, particularly if a similar patient presentation were to be encountered. In a 2017 publication the Society of Hospital Medicine, in conjunction with the Choosing Wisely Initiative, recommended against the use of FOBT for the diagnosis of UGIB due to lack of supporting benefit in current literature.^10^ Other authors mirror this sentiment and argue against the use of chemical and immunochemical FOBT in the ED altogether, citing a lack of evidence and suggesting inpatient management or outcomes are altered by results of occult blood samples.^10^,^16^ The majority of patients with suspected GI bleeds will undergo endoscopy regardless of FOBT results, and only one-third of patients admitted with a positive FOBT will end up with an endoscopic evaluation.^10^,^17^

## CONCLUSION

While fecal occult blood testing has been validated for its use in colorectal cancer screening, it has limited utility as a screening tool for UGIB in the hospital.^4^,^16^,^17^ It is important for the emergency clinician to understand these limitations and to recognize that current guidelines do not support its routine use as a diagnostic tool for UGIB. Given the lack of evidence that the FOBT is superior to a strong history and physical exam, and the potential downstream risk of adverse effects from unnecessary endoscopy, we advise against its routine use as a screening tool in the ED in the absence of other clinical features consistent with GI bleeding. Instead, we stress the importance of a detailed history and highlight the importance of clinicians to review patient medications both prescribed and over the counter, in addition to supplements and recent food ingestions when evaluating patients. Making front-line clinicians aware of the implications of false-positive guaiac testing is imperative to advance this understanding. Further, our case points to the need for future research to include the direct effect of synthetic food color additives on results of fecal occult blood testing
